# Bio-Based Degradable Poly(ether-ester)s from Melt-Polymerization of Aromatic Ester and Ether Diols

**DOI:** 10.3390/ijms23168967

**Published:** 2022-08-11

**Authors:** Lesly Dasilva Wandji Djouonkep, Alain Pierre Tchameni, Naomie Beolle Songwe Selabi, Arnaud Kamdem Tamo, Ingo Doench, Zhengzai Cheng, Mario Gauthier, Binqiang Xie, Anayancy Osorio-Madrazo

**Affiliations:** 1 College of Petroleum Engineering, Applied Chemistry in Oil and Gas Fields, Yangtze University, Wuhan 430100, China; 2 Key Laboratory of Drilling and Production Engineering for Oil and Gas, Wuhan 430100, China; 3 Lost Circulation Control Laboratory, National Engineering Laboratory for Petroleum Drilling Engineering, Yangtze University, Wuhan 430100, China; 4 Institute of Fine Organic Chemistry and New Organic Materials, Wuhan University of Science and Technology, Wuhan 430081, China; 5 Institute of Advanced Materials and Nanotechnology, Wuhan University of Science and Technology, Wuhan 430081, China; 6 Laboratory for Bioinspired Materials—BMBT, Institute of Microsystems Engineering—IMTEK, University of Freiburg, 79110 Freiburg, Germany; 7 Freiburg Center for Interactive Materials and Bioinspired Technologies—FIT, University of Freiburg, 79110 Freiburg, Germany; 8 Freiburg Materials Research Center—FMF, University of Freiburg, 79104 Freiburg, Germany; 9 Coal Conversion and New Carbon Materials Hubei Key Laboratory, Wuhan University of Science and Technology, Wuhan 430081, China; 10 Department of Chemistry, Institute for Polymer Research, University of Waterloo, 200 University Avenue West, Waterloo, ON N2L 3G1, Canada

**Keywords:** bio-based poly(ether-ester)s, mechanical properties, gas barrier properties, (bio)degradation

## Abstract

Vanillin, as a promising aromatic aldehyde, possesses worthy structural and bioactive properties useful in the design of novel sustainable polymeric materials. Its versatility and structural similarity to terephthalic acid (TPA) can lead to materials with properties similar to conventional poly(ethylene terephthalate) (PET). In this perspective, a symmetrical dimethylated dialkoxydivanillic diester monomer (DEMV) derived from vanillin was synthesized via a direct-coupling method. Then, a series of poly(ether-ester)s were synthesized via melt-polymerization incorporating mixtures of phenyl/phenyloxy diols (with hydroxyl side-chains in the 1,2-, 1,3- and 1,4-positions) and a cyclic diol, 1,4-cyclohexanedimethanol (CHDM). The polymers obtained had high molecular weights (*M*_w_ = 5.3–7.9 × 10^4^ g.mol^−1^) and polydispersity index (Đ) values of 1.54–2.88. Thermal analysis showed the polymers are semi-crystalline materials with melting temperatures of 204–240 °C, and tunable glass transition temperatures (T_g_) of 98–120 °C. Their 5% decomposition temperature (T_d,5%_) varied from 430–315 °C, which endows the polymers with a broad processing window, owing to their rigid phenyl rings and trans-CHDM groups. These poly(ether-ester)s displayed remarkable impact strength and satisfactory gas barrier properties, due to the insertion of the cyclic alkyl chain moieties. Ultimately, the synergistic influence of the ester and ether bonds provided better control over the behavior and mechanism of in vitro degradation under passive and enzymatic incubation for 90 days. Regarding the morphology, scanning electron microscopy (SEM) imaging confirmed considerable surface degradation in the polymer matrices of both polymer series, with weight losses reaching up to 35% in enzymatic degradation, which demonstrates the significant influence of ether bonds for biodegradation.

## 1. Introduction

Polymers are widely perceived as modern and versatile materials with deep roots in every sector of today’s society [1,2,3,4]. These materials have long revolutionized our daily lives by procuring numerous advantages such as performance, robustness, durability, light weight, corrosion resistance, easy processing, high productivity, low cost, and safety necessary to our needs. Over the years, among these polymers, plastics production has proliferated for short-term applications due to their facile disposal. However, they now represent permanent foreign matter in ecosystems due to their poor or lack of biodegradability, causing global white pollution [5,6]. The very versatile properties making plastics materials of choice for daily products have led to a major resource crisis linked to the exploitation of fossil fuels for the production of most common plastics [7,8]. Fortunately, the concept of sustainable development is not a recent issue; industrials and academics across the globe are deeply invested in proposing new strategies to promote this concept. This combines a dual objective: the use of renewable sources (biomass) instead of fossil ones, and the development of a low carbon bio-economy, geared toward reducing waste and minimizing resource consumption [9,10,11,12,13,14,15]. A bio-economy has the potential to serve sustainability, as it involves various components such as reuse and recycling via green routes, which favors the development of sustainable and cost-effective synthetic routes for the preparation of bio-based aromatic polyesters. In this context, biomass is an enormous source of inexhaustible polysaccharides and organic monomers with the ability to reduce the carbon footprint or monomer transformation waste in the preparation of bio-plastics [16,17,18,19,20,21]. Therefore, it is imperative for novel technologies and economic development to intercept with our society needs, without endangering the environment and its habitat, to achieve a more symbiotic ecosystem for future generations.

**Figure 1:**
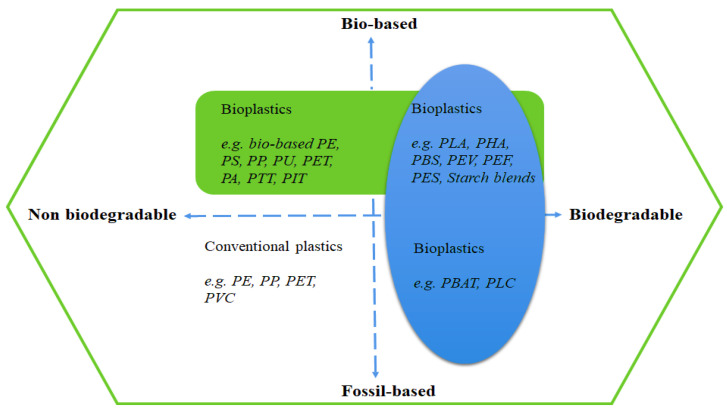
Schematic illustration of the differentiation and definition of biodegradable plastics.

Plastics are either natural, synthetic or semi-synthetic chemical compounds of high molar mass, formed by long carbon chains that can be molded into soft, rigid or slightly elastic materials [22,23]. Petro-plastics such as polyethylene (PE) [24], poly(ethylene terephthalate) (PET) [25], polyurethanes (PU) [26], polystyrene (PS) [27], polypropylene (PP) [28] and poly(vinyl chloride) (PVC) [29] are widely used in industrial and domestic applications due to their versatility and malleability. Unfortunately, they are insensitive to degradation by natural pathways, which eventually hinders the path to a bio-economy. Moreover, the evolution rate of social habits is quite slow, despite multiple policies and campaigns implemented by governments and protection agencies aimed at controlling and reducing the consumption and disposal of aftermarket plastics. Recent studies on petro-sources of chemicals used for the production of plastic packaging for consumable goods, such as ethylene, vinyl chloride, terephthalic acid and bisphenol-A, have reported that they have harmful effects on human health by promoting cancers, endocrine diseases, birth defects and many other ailments [30,31,32,33,34]. This is because these chemicals are not completely covalently bonded to the polymer matrix, so they can be released easily at all stages of the plastics’ life-cycle either via migration into liquid or solid environments or via volatilization [17,35,36,37]. The selective design and transformation of renewable feedstocks for the preparation of novel monomers/polymers remains a challenging task. At times, the terms used to describe plastics can be confusing to those not fully familiar with their nature, as some synthetic polymers are derived either from non-renewable fossil fuels or bio-based renewable sources, while others are derived from both (Figure 1). Furthermore, some polymers derived from fossil fuels blended with bio-based monomers can undergo biodegradation, while others produced from bio-based renewable sources may not. Bioplastics are essential to achieve a sustainable bio-economy, due to their huge potential to decouple economic growth and resource depletion with minimal environmental impacts.

**Figure 2:**
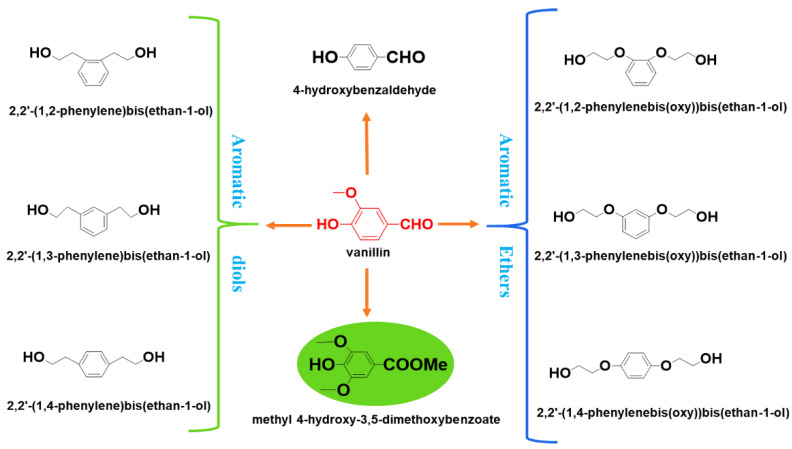
Bio-sourced monomers extracted from lignin.

Vanillin belongs to the class of benzaldehydes, with a structural formula being 4-hydroxy-3-methoxybenzaldehyde [38,39]. It is derived from polysaccharides, starch or lignocellulose [40,41] and has become a chemical of interest owing to its structural similarity to terephthalic acid (TPA), making it potentially capable of mimicking properties of polymers such as PET, poly(butylene terephthalate) (PBT) or poly(trimethylene terephthalate) (PTT) [24,25,26]. Industrially, while vanillin is mostly produced via the petrochemical-based catechol-guaiacol ‘*Solvay-Rhodia*’ process, it is also produced from lignin by the Borregaard Corporation, the second largest vanillin producer in the world, via a redesigned ultrafiltration technology using a *vanillin-from-lignin* process with minimal stream volume waste [42,43,44,45,46,47]. In this process, cellulose in wood undergoes thermal degradation to yield three significant aromatic compounds, namely anisaldehyde (3-methoxybenzaldehyde), vanillin (3-methoxy-4-hydroxybenzaldehyde) and syringaldehyde (3,5-dimethoxy-4-hydroxybenzaldehyde), alongside their diol compounds [44,48,49,50] (Figure 2).

Designing sustainable PET mimics from bio-based sources requires large amounts of aromatic components [44], and various types of polymers derived from vanillin and syringaldehyde have been reported, such as polyesters [42,43,51] epoxy resins [52,53] and polycarbonates [42,51,54,55]. For instance, Mialon et al. [44] developed a series of polyesters from polydihydroferulic acid (PHFA) that could mimic the structure and thermal properties of PET, possessing T_g_ and T_m_ values of 73 °C and 234 °C, respectively. They further investigated a series of polyesters derived from 4-hydroxybenzoic acid, vanillic acid and syringic acid, where the aromatic acids were modified with ω-chloro-alcohols on the aromatic hydroxyl group to afford hydroxy-carboxylic acids that could easily polymerize in the presence of antimony trioxide (Sb_2_O_3_) as catalyst [56]. Their study clearly demonstrated that the thermal properties associated with these polyesters are directly related to the number of aromatic methoxy substituents attached to the phenyl rings and the number of carbon atoms in the alkyl segments [17,18,57]. Similarly, Gioia et al. [43] reported a one-pot synthesis of poly(ethylene vanillate) PEV from vanillic acid in the presence of ethylene carbonate catalyzed by dibutyltin oxide, which afforded higher T_m_ than that reported by Malon (264 °C vs. 239 °C), but showed a decrease in T_g_ for increasing alkyl chain lengths. Unfortunately, these polyesters exhibited low molecular weights (ca. 5 × 10^3^ g.mol^−1^) and were practically insoluble in most solvents. Nguyen et al. [58] reported the copolymerization of 4-hydroxyethylvanillic acid with ε-caprolactone and L-lactide, achieving properties similar to those reported by Malon’s group, but still with low molar masses. Despite the fact that Pang et al. [59] successfully synthesized two series of bio-based poly(ether-ester)s from vanillic acid and linear α,ω-diols with high molecular weights of about 7.9 × 10^4^ g.mol^−1^, most of these polyesters still exhibited low processing temperatures (usually below 200 °C), low moduli, low crystallization rates and poor nucleation densities. They also lacked flexibility due to limited rotational motions around the polymer axis, which strongly hindered their utilization. To solve this problem, we inserted aliphatic cyclic diol moieties as spacers via a copolymerization strategy to control backbone flexibility and impact strength, which boosted the glass transition temperature and favored the formation of high molecular weight polyesters. The poly(ether-ester)s synthesized herein are an intriguing class of polymers, owing to the C−O−C and O−C=O bonds in their backbones. These peculiar linkages confer to the polymers a higher susceptibility to biodegradation, as the C−O−C bonds are very sensitive to acidic and basic hydrolysis or alcoholysis, as demonstrated by the Miller group [42,51].

In this study, the influence of ether and ester groups on the properties of copoly(ester-ether)s was investigated via the synthesis of two series of fully bio-based ester series (HC1, HB1, HA1) and ether series (HC2, HB2, HA2) for comparison, incorporating both aromatic and cyclic aliphatic moieties into the polymer chain. A symmetrical methylated dialkoxydivanillic diester monomer (DEMV) was synthesized from methyl 4-hydroxy-3,5-dimethoxybenzoate via direct coupling with 1,2-dibromoethane. The aromatics combine the properties of reinforced thermoplastics, while the aliphatics afford elastomeric properties via ‘*switching*’ of CHDM from *A,A*-*cis* to *E,E*-*trans* cyclohexane conformations [58]. The biodegradability of these poly(ether-ester)s was assessed under passive and enzymatic hydrolysis conditions to contribute to achieving carbon neutrality before 2050 as set by the National Council for Sustainable Development (NCSD).

## 2. Results and Discussion

### 2.1. Dimethylated Dialkoxydivanillic Diester Synthesis

**Figure 3:**
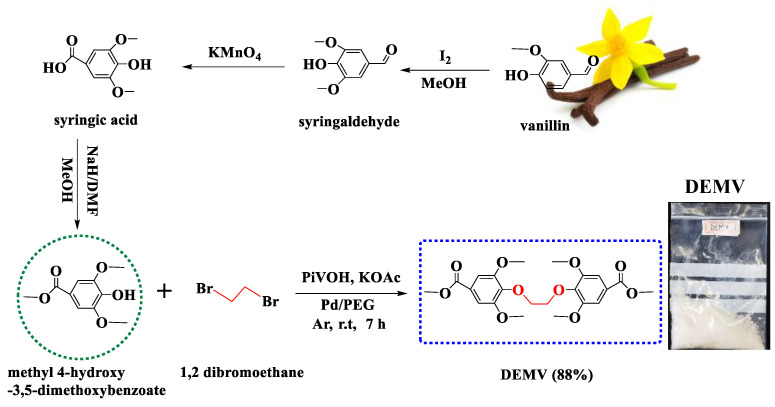
Synthesis of symmetrical biphenyl diester (DEMV).

Steam explosion (SE) is one of the most advanced, efficient and eco-friendly pre-treatment processes currently used for the production of biofuel from lignocellulose [60]. This process applied to hardwood lignin (Populus tremuloides) can yield up to 9.5% syringaldehyde, 4.7% vanillin and 0.4% 4-hydroxybenzaldehyde. Usually, the reactivity of the phenolic hydroxyl group is lower in melt polycondensation as compared to the carboxylic acid group, which often leads to unsatisfactory molecular weights. Herein, methyl 4-hydroxy-3,5-dimethoxybenzoate was first converted to dimethyl 4,4′-(ethane-1,2-diylbis(oxy))bis(3,5-dimethoxybenzoate) (DEMV), using 1,2-dibromoethane as linker with poly(ethylene glycol) palladium (Pd/PEG) as catalyst under alkaline conditions (Figure 3). This insertion of ester and aryl-ether linkages greatly enhanced the solubility of the monomer, as well as the processability and toughness of the final polymers, without substantial detrimental effects on the thermal properties of the varying substitution positions (para > meta > ortho) on the phenyl ring. The direct-coupling method offered significant advantages: dimerization of the phenolic hydroxyl groups to a diether group resulted in a symmetrical dimer, relatively easy to copolymerize with other difunctional monomers (including diols or diamines) due to its enhanced reactivity. The ^1^H NMR peaks of DEMV at δ 7.12, 4.50 and 3.83 ppm (**a**, **b**, **c** protons), assigned to the phenyl protons, methoxy protons and methylene protons adjacent to the phenoxy group, respectively, confirmed the formation of DEMV.

### 2.2. Poly(ether-ester)s by Random Copolymerization

**Figure 4:**
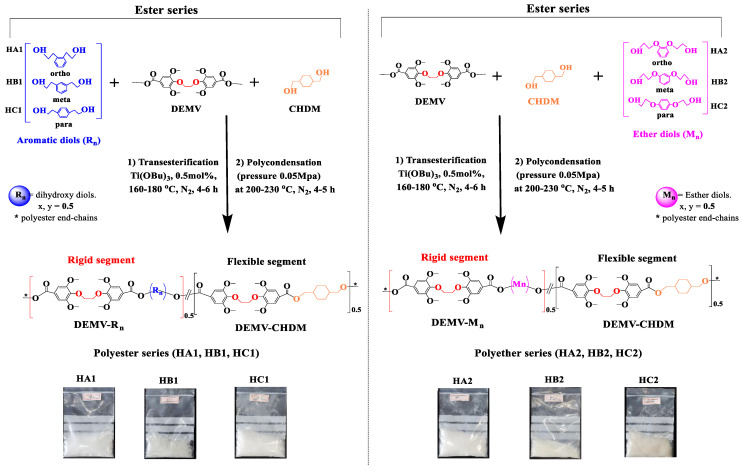
Schematic route for the preparation of poly(ether-ester)s: polyester series: HA1, HB1, and HC1; polyether series: HA2, HB2, and HC2, respectively.

The poly(ether-ester)s were synthesized as depicted in Figure 4. Dimethyl 4,4′-(ethane-1,2-diylbis(oxy))bis(3,5-dimethoxybenzoate) (DEMV) was initially reacted with aromatic diols derived from vanillin (m = 1,2; 1,3; 1,4) via direct esterification and subsequently polymerized under the conditions specified in Table 1. The diol-to-diester mole ratios used in the reactions were slightly higher than one to ensure that the polymer chains were terminated with hydroxyl groups. The excess of diol used (including Rn; Mn and CHDM) also compensated for diol loss by evaporation during Step 2. Elevated temperatures and long reaction times were necessary in both stages to achieve complete reactions, so as to obtain polymers with high molecular weights. In the 2nd step, the viscosity increased rapidly under reduced pressure, and the colorless mixtures gradually transitioned to a whitish-light brown coloration at temperatures exceeding 230 °C. All of the copolymers displayed significant solubility in chloroform, appearing as white semi-crystalline solids after purification, allowing the evaluation of their molar mass by gel permeation chromatography (GPC) analysis. Samples HC1 and HC2 had the highest *M*_w_, possibly due to their optimal spatial orientation (para-substitution) for the esterification reaction, which reduced steric hindrance exerted on the polymer ring. For comparison, Kurt and Gokturk [61] prepared a series of polyesters with good thermal stability from ethyl vanillin, vanillic acid, and syringaldehyde. However, these polyesters had relatively low molar masses of only 0.8–1.3 × 10^4^ g.mol^−1^ due to their extreme rigidity. It can thus be concluded that the insertion of an aliphatic chain was beneficial to achieve high molecular mobility and molecular weights. This result can be explained by considering the fact that aliphatic rings positively impact the thermal and mechanical stability of poly(ether-ester)s, displaying better performance than the corresponding terephthalic aromatic polymers and furan-derived analogues [51,54,55]. These attractive properties are intermediate between the two homopolymers (DEMV-Rn > DEMV > DEMV-CHDM). Nevertheless, the molar masses of the poly(ether-ester)s appear to meet the requirements for most packaging industry applications (5.27–7.88 × 10^4^ g.mol^−1^). The Mark–Houwink intrinsic viscosity [η] constants were measured by capillary Ubbelohde viscometer (Schott Gerate GMBH) at 25 °C in a mixture of phenol and tetrachloroethane (60/40, *w/w*), and the values were found to increase gradually with the molecular weight of the samples [62,63,64].

### 2.3. FTIR and NMR Analyses

**Figure 5:**
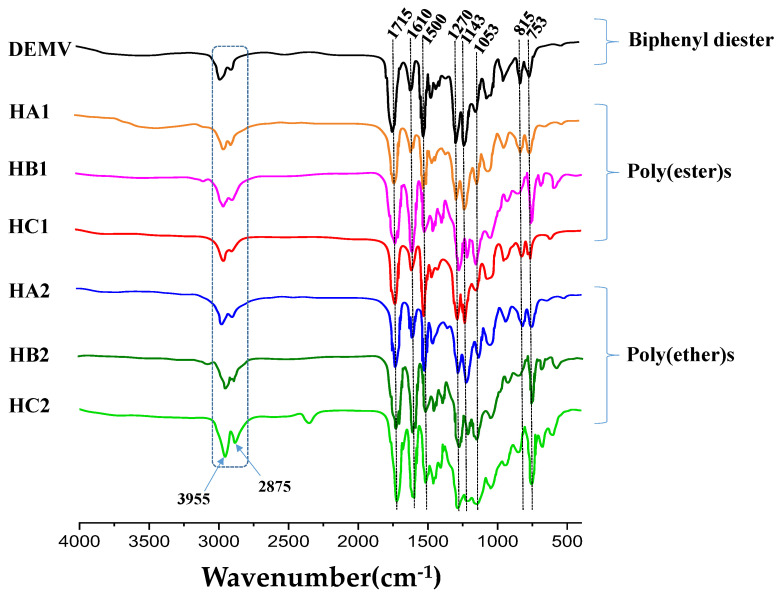
FTIR spectra of poly(ether-ester)s.

The FTIR spectra of the poly(ether-ester)s shown in the Figure 5, displayed similar characteristic absorption bands. This included weak asymmetric and symmetric C-H bending vibrations at 2955 and 2875 cm^−1^, respectively, and a strong C=O peak attributed to the stretching vibration of ester carbonyl group attached to the phenyl ester at 1715 cm^−1^. The absorption bands at 1600 and 1500 cm^−1^ were attributed to the disubstituted aromatic segments (C=C bond stretching) of the polymer chains [50,65]. A distinctive absorption peak was observed for all of the ether series at 1270 cm^−1^ (sp^2^), attributed to C–O alkyl ether stretching. Additionally, two sharp signals at 1180 and 1143 cm^−1^ suggested the presence of methoxy groups attached to the phenyl rings, while a C–O (sp^3^) stretching absorption peak appeared at 1053 cm^−1^. Interestingly, the absorption peaks at 815, 753 and 620 cm^−1^ described in-plane bending vibrations of unsaturated C–H bonds on the meta, para and ortho-disubstituted phenyl units. Therefore, the characteristic peaks for the phenyl, ether and ester groups in the polymer structure strongly supported the successful incorporation of all of the monomer components.

**Figure 6:**
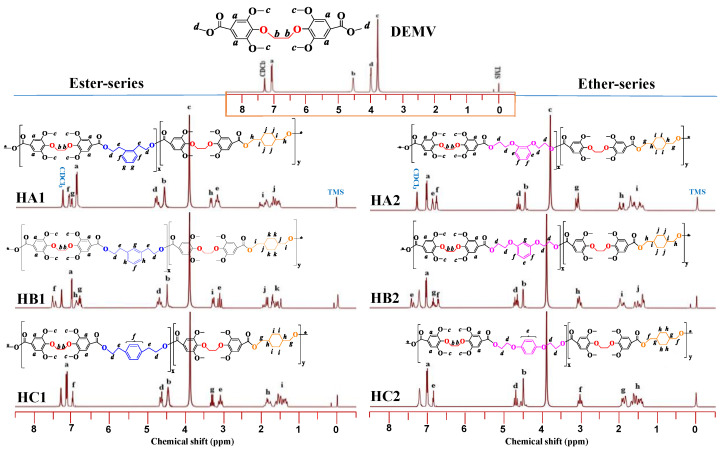
Chemical structure and ¹H NMR spectra of poly(ether-ester)s.

The ¹H NMR spectra and corresponding chemical structures of the synthesized poly(ether-ester)s are provided in Figure 6. From the ^1^H NMR spectra, the disappearance of the methyl ester signal for protons (**d**) in DEMV strongly supported the formation of long chain copolymers. For DEMV units, characteristic peaks were observed at 7.12 ppm (**a**) and 4.50 ppm (**b**). The signals observed in the 7.19–7.15 ppm (**f**, **g**) and 6.98–6.84 ppm (**e**, **f**) intervals corresponded to the HA1 and HA2 protons on the meta-disubstituted phenyl ring, respectively. Similarly, the signals at 7.40, 6.85–6.93 ppm (**f**, **h**, **g**) and 7.39, 6.38–6.61 ppm (**e**, **g**, **f**) matched the signals for HB1 and HB2 on the ortho-disubstituted phenyl ring, while the signals at 6.81 (**f**) and 6.98 (**e**) ppm corresponded to the para-disubstituted protons on the phenyl ring. For the ester series, the signals corresponding to the methylene group adjacent to the ester bonds were sensitive to orientation effects and split into multiple peaks (3.12–4.60 ppm, **d**, **e**) due to their unsymmetrical nature, whereas for the ether series, the methylene protons adjacent to the ether bond were insensitive to orientation effects and appeared as a single peak (4.55–4.60 ppm, **d**). The signal at 3.83 ppm was attributed to the methoxy groups attached to the DEMV ring. CHDM signals were observed in the range of 2.10–1.35 ppm for both series with peaks **g**, **h**, **j**, **k** and **i**, respectively. With the exception of the solvent peak (CDCl_3_) at 7.20 ppm and the reference (TMS) signal at 0 ppm, the integrals for all of the peaks associated with each monomer were close to the feed ratios in all cases, which confirmed the successful synthesis of the desired poly(ether-ester)s.

**Figure 7:**
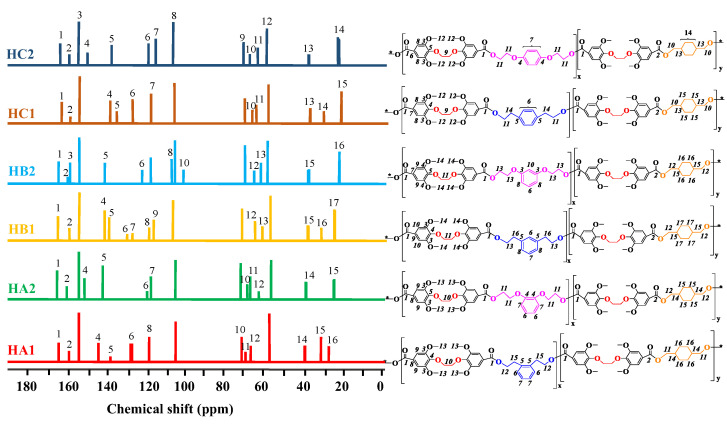
^13^C NMR spectra for poly(ether-ester)s.

As shown for the ^13^C NMR spectra in Figure 7, various signals were attributed to carbon atoms linked to different functional groups in the poly(ether-ester)s chains. They were observed in distinctive regions associated with the various carbon functionalities present in the polymer backbone. Taking the spectrum for HC1 as an example, peaks at 164.5 and 160.1 ppm (**1**, **2**) corresponded to the quaternary carbonyl ester groups of DEMV, while the resonance signals observed at 156.2, 144.3, 120.0, and 105.6 ppm (**3**, **4**, **7**, **8**) matched the diester carbons, and the peak at 71.5 ppm (**12**) was for the methoxy carbons attached to the aromatic rings. Aromatic diol carbon peaks were observed at 130.8 and 130.1 ppm (**5, 6**), while the para-disubstituted ethyl carbons attached to the DEMV carbonyl were observed around 65.1 and 32.3 ppm (**11**, **14**), respectively. Finally, at lower ppm values, the CHDM carbon peaks linked to the diester carbonyl were detected at 65.4, 40.2, and 25.5 ppm (**10**, **13**, **15**). All of the carbon signal shifts observed were in accordance with expectations, reiterating the successful synthesis of the poly(ether-ester)s.

### 2.4. Thermal Analysis

**Figure 8:**
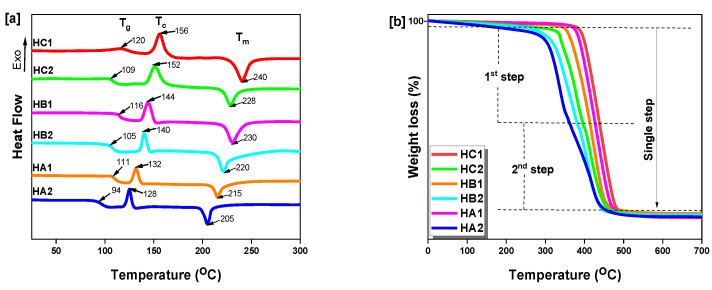

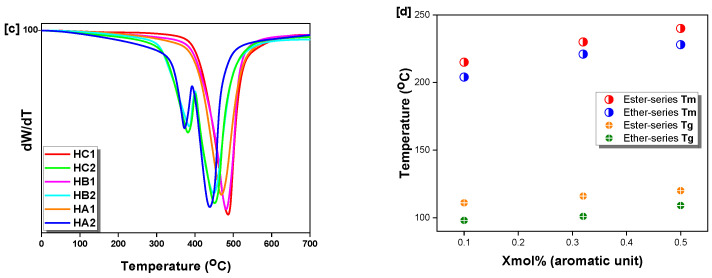
(**a**) DSC, (**b**) TGA, (**c**) DTG of poly(ether-ester)s and (**d**) relation between T_g_, T_m_ and composition.

The thermal properties of the poly(ether-ester)s were analyzed (Figure 8), and the results obtained are summarized in Table 2. The DSC curves are provided in Figure 8a, from which the melting temperature (T_m_), normalized melting enthalpy (ΔH_m_), crystallization temperature (T_c_), normalized crystallization enthalpy (ΔH_c_), and glass transition temperature (T_g_) were obtained. In polymer chemistry, morphology is a key factor distinguishing amorphous and crystalline solids. Herein, the poly(ether-ester)s displayed semi-crystalline behavior with crystallization peaks from 165–206 °C. It was noticed that variations of the dihydroxyl substituents from *para-meta-ortho* positions in the di-substituted phenyl rings influenced crystallinity, resulting in decreases in both the melting point and melting enthalpy from 240–205 °C and from 105.6–73.8 J/g, respectively. This is attributed to strong repulsive effects (steric hindrance) arising between the different monomers in space, causing a disruption of chain regularity and leading to a decrease in crystallinity, as indicated by the drops in T_c_ and peak intensity [66]. These polymers possess semi-rigid structures, where the regularity of the polymer chains varies between semi-crystalline and amorphous domains. This decrease demonstrates the existence of phases with a lower degree of order (mesophases) than the crystalline domains, in amounts directly related to the CHDM-units content. In this system, the incorporation of DEMV units along the polymer backbone increased the free volume caused by the spatial disposition of the methoxy groups on the biphenyl structure. In addition, the cyclic aliphatic rings introduced between aromatic units not only enhanced chain mobility but also boosted thermal stability (*vide infra*), whereas the T_g_ increased in each series. This cyclic unit, when exceeding a certain level, caused a considerable decrease in resistance to motions during its *cis-to-trans* conformation transitions. By so doing, amorphous domains were introduced in the polymer chains, owing to a considerable number of chain loops sticking out of orderly crystalline domains, much like wild hairs sticking out from a nicely groomed hairdo [67]. These polymers could crystallize upon heating in DSC analysis because of their fast crystallization rate, attributed to the aromatic species (DEMV–H), while the DEMV–CHDM unit inserted inside the DEMV–H-type units retained a larger proportion of amorphous character. Even though esterification is a convenient method to modify or tune vanillin-derived monomers and achieve thermally stable diesters, the ether linkage rather tends to decrease the T_g_, as depicted in Table 2. However, all of the copolymers maintained satisfactory T_g_ values and melting points when compared to existing vanillin-derived polyesters and their analogous petro-chemical counterparts such as PET. HC1 had a T_g_ reaching 120 °C, which suggests that the crystallinity level of that sample was superior to PEV [55] and PET [68] homopolymers. The copolymer compositions leading to high chain mobility promoted the alignment of their long-chain structure, which induced rapid crystal growth at elevated temperatures (T_c_ = 156 °C), as observed during the cooling scan from the melt. Studies performed by the Berti group attributed partially amorphous phases generated by CHDM after melt quenching to the *trans*-CHDM units, for which crystalline domains were only formed when the aromatic content exceeded 50% [69]. The results obtained confirm that crystallization occurred successfully for almost equivalent insertion of *diester-to-diols* units (approximately 52:48 mol%). Comparison of the poly(ether-ester)s with other vanillin-based polymers (PE-ms and PEV) suggests that the enhanced thermal properties are due to the good affinity of the aromatic aliphatics, as seen in Table 2.

In the TGA and dTG curves (Figure 8b,c), the ether series displayed a slight double-transient region of thermal decomposition, as compared to single-step smooth decomposition curves for the ester series. This dual decomposition region may be due to the rupture of the C–O–C ether bonds, followed by carboxylic ester group [–O–C(=O)–O–] and C–C bond cleavage. The onset decomposition temperatures (5% weight loss) were in the range of 315–430 °C, which demonstrates that the materials possess a broad thermal processing window, while the decomposition temperature at 50% (T_d, 50%_) ranged from 412–445 °C, again confirming the good thermal stability of the copolymers. The ester series had higher T_g_ values and thermal stability as compared to the ether series, due to the absence of intrachain ether bonds readily cleaving at elevated temperatures. The char residue for the poly(ether-ester)s at 700 °C was less than 9%, demonstrating an appreciable extent of thermal decomposition suitable for thermal recycling and reprocessing.

The variations in T_g_ and T_m_ are depicted in Figure 8d. It is interesting to note that the T_g_ increased steadily with disubstitution from *ortho-to-para*, and with composition from 0–50 mol% of phenyl rings for both series, and similarly for T_m,_ even though this effect was more pronounced in the ester series. A possible explanation for this lies in the repeat distance measured by molecular chain modeling [70,71], which showed that polymers containing internal oxygen-rich functional groups have longer repeat chain units than polyesters. Consequently, the ester series displayed enhanced thermal properties and symmetry per unit distance along the polymer chain compared to the analogous ether series.

### 2.5. Mechanical and Dynamic Testing

**Figure 9:**
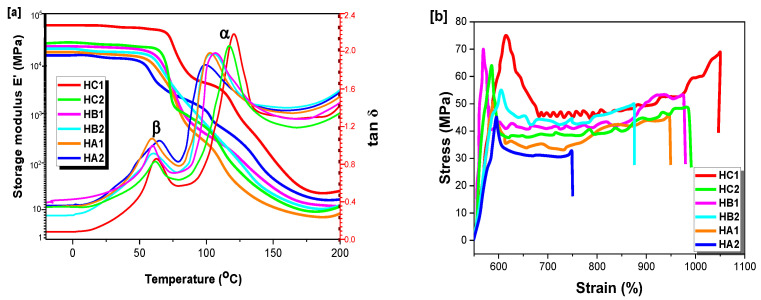
(**a**) Dynamic mechanical thermal analysis (DMTA) of the poly(ether-ester)s, showing the evolution of the storage modulus E’ and tan δ with temperature. (**b**) Tensile testing curves for the poly(ether-ester)s.

The rheological properties of compression-molded poly(ether-ester)s samples, as determined by dynamic mechanical thermal analysis (DMTA), are compared in Figure 9a. The stress–strain diagrams obtained by tensile testing are also shown in Figure 9b. Table 3 provides a summary of the Young’s modulus (E), tensile strength (σ_b_), and elongation at break (ε_b_) determined.

For a perfectly elastic solid, the strain and stress are in phase, but a time shift between the two signals increases in magnitude as the viscous character of the sample increases [72]. This enables the calculation of the storage modulus and loss tangent (tan δ) for the samples (Figure 9a). The main transition, known as the α-transition or the glass transition temperature, is defined as the temperature of the peak maximum. Comparison of the T_g_ values determined by DSC analysis (Table 2) with the DMTA curves of Figure 9a shows a rough correspondence of the transition temperatures determined by both methods, but with significant discrepancies. Indeed, the T_g_ of semi-crystalline polymers is sensitive to their crystallinity level, and, therefore, to the thermal history of the samples. Since it is easier to control the thermal history of the samples in DSC analysis than for compression-molded DMTA samples, the DSC results are considered more reliable. The second largest transition (β-transition) is typically correlated with the occurrence of side-chain or functional group motions in polymers. While the clear identification of the origin of the β-transition would require a more in-depth investigation, it could be related to the occurrence of chair-chain flip transitions of the cyclohexyl units in the polymer chains.

To investigate the influence of ether-ester bonds on the mechanical properties, the tensile test results can be compared with those obtained for vanillin-based polymers such as PE-ms, furan-based PBFGA and conventional PET, synthesized by Zamboulis [55], Pang [59] and Celik [68], respectively. The stress–strain curves for the poly(ether-ester)s are provided in Figure 9b, and the mechanical parameters extracted from them are listed in Table 3. The ester series, containing no ether bonds, had a better mechanical performance than the ether series. Namely, the tensile modulus and the elongation at break both decreased for the ether series. However, the effects were less important for HC1, which exhibited an elastic–plastic behavior with stable crack growth when stretched. This might be related to the presence of the CHDM units, which may behave not as reinforcements but rather as defects in the matrix, as compared with the higher tensile strength observed for the parent homologues (DEMV-Mn) due to the single species of repeating units present. The more symmetrical *para*-substituted phenyl units in HC1 conferred a higher degree of crystallinity to the chains, which led to the best tensile behavior among the samples. The poly(ether-ester)s exhibited a tensile strength of 44–75 MPa (Figure 9b), slightly below PET (84.8 MPa), but had an elongation at break up to 12.7-fold higher (1052%) than PET (82.7%). When comparing HC2 and HB1, one can observe that despite the presence of ether linkages in HC2, the elongation at break was still 1.5-fold higher, presumably due to low steric hindrance exerted by the linkages in the *para*-position of the phenyl as compared to the *meta*-position of HB1. These interesting results explicitly demonstrate that poly(ether-ester)s derived from syringaldehyde, itself derived from vanillin, possess great potential as replacement materials for commercial petrochemical-based polymers.

### 2.6. Barrier Properties of Poly(ether-ester)s

Permeability and transparency are both very important parameters in the packaging industry for the protection they provide in terms of product quality and durability. These barrier properties need to match the sensitivity of the food/beverages and their specified shelf life [74]. For example, dairy foodstuff that produces CO_2_ (such as fermented milk) requires packaging allowing the permeation of CO_2_, while simultaneously protecting it from O_2_, which eventually can oxidize fat. As a result, food-packaging products require a sophisticated protection mechanism to extend their shelf life, while maintaining the desired quality and characteristics. However, these parameters depend on several factors such as the shape and size of the matrix and its orientation relative to the direction of permeation, defined as the direction in which a penetrant passes through a polymeric material [75]. Many commodity polymers possess limited effectiveness against gas or liquid permeability. The rate of gas permeability through a polymer matrix is determined by two factors: (a) the solubility of the penetrant in the polymer, and (b) the relationship between the size of the gas molecules and the interstices present in the polymer matrix. Taking into account this complex scenario, an O_2_ barrier improvement factor (BIFp) was defined by Burgess et al. [76] as the ratio of O_2_ permeability coefficient of PET vs. the O_2_ permeability coefficient of the target polymer. The permeability coefficients of O_2_ and CO_2_ were determined at 23 °C (Table 4), using films prepared by compression molding and stored at room temperature for 5 days.

**Figure 10:**
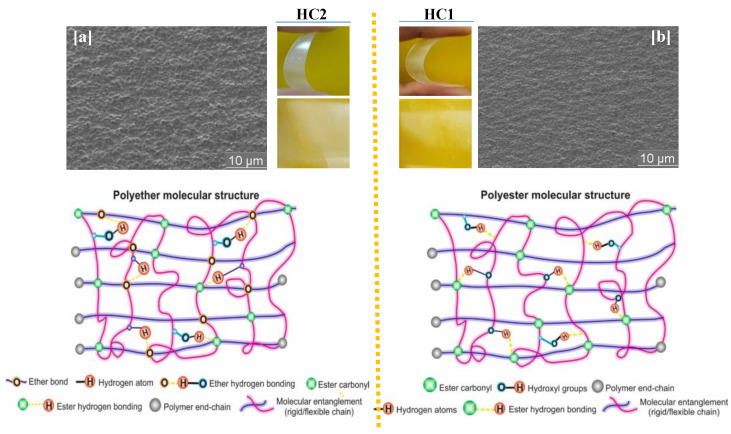
Schematic representation of the molecular structure and permeability differences in poly(ether-ester)s. SEM micrographs of HC2 (**a**) and HC1 (**b**) polymers.

The permeability of a gas or a liquid across a polymer matrix is nevertheless a complex phenomenon encompassing several factors ranging from modeling to the interpretation of the experimental data. These factors include the length-to-diameter ratio, chain orientation and film disparity, the shape, density, free volume and crystallinity of the matrix, as well as the affinity between the constituent polymers and the diffusing species [77]. Consequently, differences in molecular structure, composition and chain regularity among the different types of copolymers resulted in varying barrier properties. The morphology of these multiphase (semi-crystalline) systems affected the diffusion path of the permeating molecules, the degree of entanglement of the chains, and the free volume of the samples [77,78]. From previous studies, aromatic-aliphatic polyesters derived from biomass, such as thiophenedicarboxylic acid, dimethyl carbonate, vanillin and lactic acid, present excellent barrier properties in comparison with polyolefins such as polyethylene (PE), due to their ring structures leading to properties analogous to terephthalic acid (TPA) [79,80,81,82]. As a result, the poly(ether-ester)s displayed satisfactory barrier properties not only due to their structural mimics, but also due to the short chain length of the DEMV diester with the methoxy groups, which preserved the rigidity of the copolymers. Interestingly, the ether-series samples, possessing internal ether bonds, displayed outstanding barrier properties when compared to ester-series samples, presumably due to the ability of these ether bonds to form hydrogen bonds similarly to the well-known carbonyl ester groups. These results are in agreement with recent studies stating that increased hydrogen bonding considerably improves the gas barrier properties at low or intermediate relative humidity levels [83,84]. That is, the presence of C–H–O and C–O–C interactions between adjacent copolymer chains is highly effective at preventing gas molecule migration, since the gas molecules moving across smaller polymer pores are intercepted, resulting in low permeation and diffusion coefficients, as seen from the diffusion mechanism and SEM surface morphology depicted in Figure 10. Crystallinity also affects permeability, since an orderly, tightly packed polymer matrix prevents gas permeation better than amorphous regions. Consequently, orientation patterns on the film surface may have greatly contributed to the excellent barrier properties exhibited by the poly(ether-ester)s. That is, changes in disposition of the di-substituted phenyl rings (*ortho-to-meta-to-para*) strongly improved the gas barrier properties by increasing molecular chain packing, while also decreasing the free volume in the polymers. The excellent barrier performance of HC1 is attributed mainly to its semi-rigid structure, high molecular weight, and relatively higher T_g_. In comparison, the homopolymers (DEMV-Mn and DEMV-CHDM) exhibited a higher transparency, especially DEMV-HC1 (*para*-oriented), but displayed poorer gas barrier properties. It was demonstrated in several studies that the presence of inter- and intramolecular hydrogen bonds greatly influences the barrier properties, to the same extent as film morphology and molecular size. As compared with TPA, the symmetric structure of the biphenyl diester promoted the rotational conformation changes of the rings, which enhanced the barrier properties of the poly(ether-ester)s. With variations in aromatic content, the CO_2_ barrier performance of poly(ether-ester)s was 1.8–2.6 times lower, while the O_2_ barrier coefficient was 3.2–9.5 times lower than that for PET, but slightly higher than that for PEF. These permeability coefficients were compared to PET as one of the most commonly used packaging materials.

### 2.7. Degradability Studies

**Figure 11:**
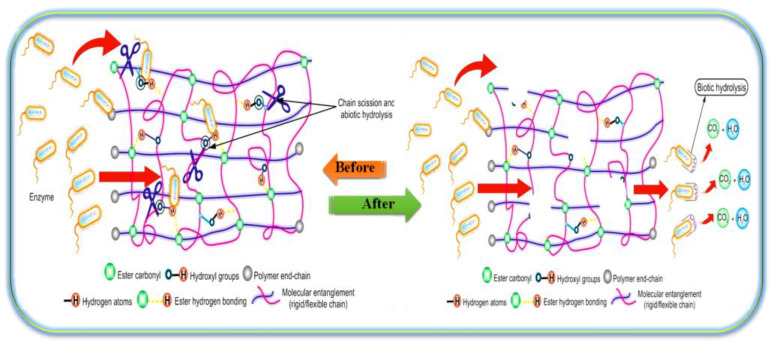
Degradation mechanisms of poly(ether-ester)s.

Polymer degradation involves different processes such as physical, chemical, and biological routes or a combination thereof, under the influence of several factors such as temperature (thermal degradation), air (oxidative degradation), water (hydrolytic degradation), microbial (biodegradation), light (photo-degradation), high-energy radiation (UV, γ-irradiation), chemical agents (corrosion), and mechanical stress [86,87,88]. These factors lead to irreversible changes in the materials and play a major role in the colonization by microbes and biofilm formation. This process usually occurs in two stages: deterioration of the appearance, such as changes in color, physical and morphological properties (disintegration), and eventually the release of metabolic by-products (mineralization) such as water, carbon dioxide, and other simple inorganic compounds. Thus, molecular chain scissoring can be initiated either via passive hydrolysis or enzyme-catalyzed hydrolysis. However, this type of degradation mostly depends on the type and nature of the bonds present within the polymer backbone. While degradable polymers contain labile bonds, these bonds at times appear to be very stable under physiological conditions and, therefore, require enzymatic catalysts to undergo biodegradation, as shown schematically in Figure 11 [46,50].

**Figure 12:**
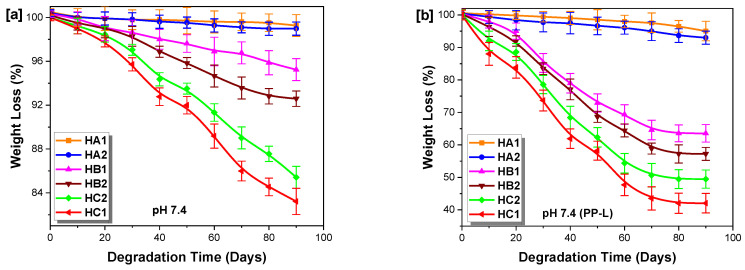
Residual weight loss vs. incubation time for poly(ether-ester)s under different conditions: (**a**) without PP-L, (**b**) with PP-L.

In this work, passive hydrolytic degradation was studied in aqueous phosphate buffer solutions, while enzyme-catalyzed hydrolysis was achieved in the same buffer solutions with 0.1 mg/mL of *Porcine pancreas lipase* (PP-L) for 90 days, both at pH 7.4 and 37 °C. The degradation kinetics curves (Figure 12) indicated that the rate of weight loss for the poly(ether-ester)s was greatest in the enzymatic solutions.

In passive hydrolysis (Figure 12a), the degradation rate relied on two factors: the vulnerability of the labile bonds to water hydrolysis (permeability), and the amount of free volume within the polymers. The change in weight was relatively small, which was attributed to the labile bonds being shielded by the hydrophobic structure, which eventually lowered the effective rate of hydrolysis. The degradation proceeded slowly, with little or no weight loss observed within the first 20 days and increasing later, corresponding to the permeation time needed to weaken the labile bonds. Furthermore, the high molecular weight of the copolymers had no direct influence on the hydrolysis rate. During the initial stages, the films lost 1–2% of their mass, and only after 32 days was there an appreciable weight drop. Samples HC2 and HC1 displayed satisfactory weight losses from 7.5 to 9%, respectively, while samples HB1 and HB2 displayed minor losses of less than 5 wt% over 35 days, and samples HA1 and HA2 displayed minimal weight losses (< 3 wt%), perhaps due to enhanced steric hindrance of the di-substituted phenyl rings.

In enzymatic degradation (Figure 12b), the enzymes can depolymerize the poly(ether-ester)s into building blocks or monomers for further polymer production and effective recycling of the materials into equal- or possibly higher-value components. The rupture of the labile bonds is orchestrated by the growth of microbes on the plastic surface, which affects the mechanical properties and leads to the formation of macro-oligomers that migrate from the polymer matrix into the degradation medium to be finally absorbed and mineralized by the microorganisms [89]. Likewise, minimal weight losses were observed in the early stages (<15 days) of incubation with PP-L, corresponding to the promotion of microbial colonization on the hydrophobic plastic surface, essential for abiotic hydrolysis, to reduce polymer buoyancy and hydrophobicity. The randomly cleaved labile bonds then formed shorter chain segments with the help of modifying/hydrolyzing microbial enzymes. It was noticed that the rate of degradation was directly related to the rate of diffusion and permeation coefficient, with water diffusing into the matrix relatively faster, and then initiating chain cleavage, as reported by Bu et al. [90]. In addition, the types of functional groups present (ester-carbonyl, ether bonds and hydroxyl), together with the molecular weight, greatly influenced the degradation trends. Herein, the ether series displayed higher weight losses due to the hydrolysis of their labile ether, ester and hydroxyl bonds, as compared to the ester series, with only ester and hydroxyl groups. Interestingly, HC1 exhibited the highest degradation rate via chain scission in the backbone, possibly due to its high permeability and molecular weight (Figure 10). As monitored by GPC, HC1 had a satisfactory decrease in molar mass from 78,800 to about 45,700 g/mol after only 60 days of incubation. Chain cleavage reduced the mobility of the polymer chains, causing them to gradually crystallize. In general, for all of the series, a decrease of 15–35% in molar mass was observed, with the exception of HA1 and HA2 possessing considerable steric hindrance. Increases in polydispersity indices were observed from 2.18–3.15 after 90 days of incubation, which is attributed to the growth of the low mass fraction, confirming the existence of chain-scission occurring in the polymer matrix. The number-average molar mass of the poly(ether-ester)s decreased as well, as demonstrated by Siracusa et al. [91] and Mohanan et al. [92], who explained that the degradation process occurred near the chain-ends by selective enzymatic hydrolysis, but also for any accessible labile bonds in the polymer matrix. The significant weight loss and decrease in number-average molar masses indicate the low retention rate of the aromatic chain fragments, which would be suitable as safe and convenient eco-friendly packaging films. The enzymatic degradation rate thereby followed the order of HC1 > HC2 > HB2 > HB1 > HA2 > HA1.

**Figure 13:**
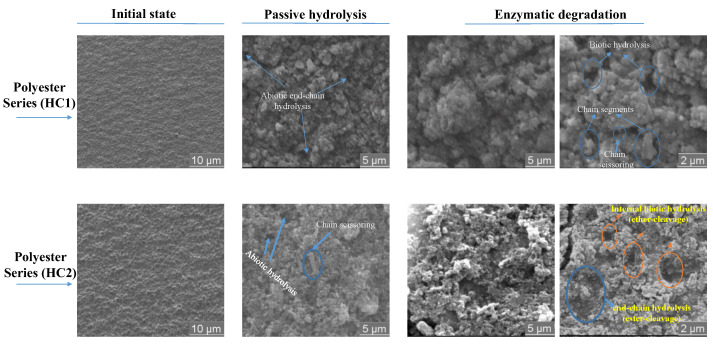
Enzymatic degradation and visual observation of degraded films after 90 days of incubation at a constant pH of 7.4.

The degradation process was also investigated at the microscopic level, using scanning electron microscopy imaging (SEM) (Figure 13). In the case of passive hydrolysis, the recovered samples displayed minimal morphological changes (both for the ester and ether series), presumably due to inaccessibility of the ester carbonyl and ether groups, as a result of high steric hindrance of the di-substituted phenyl rings acting as hydrophobic moieties. In contrast, significant morphological changes were detected for all of the poly(ether-ester)s under enzyme-catalyzed hydrolysis, which exhibited multiple cleaved zones on the entire polymer surfaces. Thus, enzymatic degradation proved to be an effective method, with enormous potential for the monomers to be recycled and re-used for further polymer syntheses, rather than being discarded into the environment.

## 3. Materials and Methods

All of the air- and moisture-sensitive materials were synthesized and manipulated in flasks or Schlenk-type bottles under high vacuum or nitrogen (N_2_) atmosphere.

### 3.1. Materials

Methyl 4-hydroxy-3,5-dimethoxybenzoate (98%), 1,2-dibromoethane (99%) and 1,4-cyclohexanedimethanol [CHDM], and mixture of *trans*- and *cis*-isomers (*trans/cis* = 95/5, 98%) were obtained from the Sinopharm Chemical Reagent Co. The aromatic diols (98%) were obtained from Zhengzhou Xipaike Technology Co. Potassium acetate, pivalic acid, poly(ethylene glycol) palladium (Pd/PEG), and Ti(OBu)_3_ (99%) were purchased from the Aladdin Reagent Co. (Shanghai, China), while chloroform, methanol, dichloromethane, hexane and N,N-dimethylformamide (all analytical grade) were purchased from the Sinopharm Group. All of the chemicals were used without further purification.

### 3.2. Characterization Techniques

FTIR spectroscopy was performed on a Bio-Rad FTS6000 spectrophotometer at room temperature in the range of 400–4000 cm^−1^, at a resolution of 0.4 cm^−1^ with 80 scans, averaging six spectra. The polymer samples were prepared as KBr pellets.

NMR spectra were recorded in CDCl_3_ on a Bruker AC-400 NMR spectrometer (400 MHz for ^1^H NMR and 101 MHz for ^13^C NMR), with 0.03% (*v/v*) tetramethylsilane (TMS) as internal standard at room temperature. The characteristic peaks were assigned and integrated to determine the mole fraction of copolymer constituents.

The number-average molecular weight (M_n_) and dispersity (Đ = *M*_w_/M_n_) were measured by GPC on a Shimadzu 10A-VP system. All measurements were carried out at 40 °C using chloroform as the eluent at a flow rate of 1.0 mL/min, with polystyrene gel columns (Shimadzu Simpack GPC-80MC × 2 and GPC-8025C) and polystyrene standards.

DSC analysis was performed on a DSC 8500 instrument (PerkinElmer). A sample size of 5 mg was used. The heating was executed stepwise with heating–cooling–heating cycles, from 25 to 300 °C at a heating rate of 10 °C/min. The glass transition temperatures (T_g_) and melting point (T_m_) values were obtained from the second heating cycle, at a heating rate of 10/min and under N_2_ atmosphere. The enthalpy of melting (∆H_m_) was calculated by integration of the normalized area of the exotherm. The degree of crystallinity was also determined and derived from the ratio of the melting enthalpy to the heat of fusion of the semi-crystalline polymers (∆H_c_).

TGA was performed on a Seiko Exstar 6000 TGA quartz rod microbalance under N_2_ atmosphere. The samples were heated from 25 to 700 °C at a rate of 10 °C min^−1^ (gas flow 40 mL min^−1^). The decomposition temperature at 5% weight loss (T_d,5%_) and the temperature of maximum decomposition rate (T_d, max_) were assessed. The samples (8 ± 0.2 mg) were placed in aluminum crucibles, while a blank measurement was performed and subtracted from the experimental curve to eliminate the buoyancy effect.

After grinding, samples were compression-molded on a Model 3889 Hot Press (Carver Inc., Ontorio, NY, USA), and rheological testing was performed on a dynamic mechanical analyzer (TA Instruments DMA Q800, Waters Technology Co., Ltd., Shanghai, China) with a preload force of 0.1 N, an amplitude of 10 µm, and a frequency of 1 Hz at 25 °C. Samples (15 g) were molded in a 3.500 by 3.500 mm^2^ window mold (1.8 mm thickness) at 140 °C and 7500 PSI pressure into dogbone-shaped samples for 45 min, then cooled for 15 min at 25 °C and released from the mold. To characterize further the mechanical properties, tensile tests were performed in triplicate, with the average values recorded for the tensile strength, elongation at break and tensile modulus. For mechanical analysis, each sample was cast from chloroform solutions at a concentration of 100 g.L^−1^.

The CO_2_ and O_2_ barrier properties were measured at 23 °C using a MOCON Analyzer (MOCON OX-TRAN, Model 2/21, MH module), following the *Gas Permeability Testing Manual and Standards* ASTM 1434-82 (standard test method to determine the gas permeability characteristics of plastic films and sheeting, 2009). The films with a surface area of 38.5 cm^2^ were melt-pressed, and the barrier improvement factor was calculated accordingly. The tested polymer films were placed in-between two chambers. One chamber was filled with the gas under investigation (P = 0.1001 MPa, T = 23 °C; gas stream = 100 cm^3^/min; 0 or 85% relative humidity), and the other was filled with CO_2_ gas. From the pressure/time curve, the software calculated the permeability values representing the barrier properties of the films. For all samples, triplicate measurements were taken, and the mean value was reported.

Passive and enzymatic degradation was studied on films of 0.3 mm thickness incubated in phosphate-buffered solution (pH 7.4) at 37 °C, with and without 0.1 mg/mL of *Porcine pancreas lipase* (PP-L), over a period of 90 days. PP-L was used for its high reactivity (55 units/mL) and good stability (reported by the manufacturer, Sigma-Aldrich, Taufkirchen, Bavaria State, Germany). The pH was monitored using a pH meter (Orion 420+, Thermo-Electron Corporation, Wanchai, Hong Kong, China). The enzyme solution was replaced every 3 days to maintain full enzymatic activity. Every 7 days, the films were removed from the solutions, washed gently with deionized water and methanol (to quench the enzyme), and dried to constant weight at 60 °C in a vacuum oven for 6 h. Identical conditions were used for each measurement, and the standard deviation on the measurements did not exceed 0.2%. The extent of degradation was expressed in terms of weight loss given by the equation below:

(1) $$\mathrm{Degradation}\;\mathrm{rate}\;\left(\%\right)=\frac{X_{1}-X_{2}}{X_{1}}\times 100$$

where X_1_ and X_2_ are the film weights before and after degradation, respectively.

Furthermore, the effects of incubation with and without lipase on the surface morphology of the films were evaluated by scanning electron microscope (SEM) imaging, operated on a JEOL JSM-IT500 device. The samples were coated with a thin gold layer under vacuum, and the study was carried out at 5 kV acceleration voltage.

### 3.3. Synthetic Procedures

#### 3.3.1. Synthesis of Dimethyl 4,4′-(Ethane-1,2-diylbis(oxy))bis(3,5-dimethoxybenzoate) (DEMV)

The biphenyl diester DEMV was successfully prepared from methyl 4-hydroxy-3,5-dimethoxybenzoate and 1,2-dibromoethane through a direct-coupling reaction. In a 250 mL single-mouth flask with a reflux condenser, a solution of syringaldehyde (10 g, 50 mmol), potassium acetate (0.5 g, 5 mmol), pivalic acid (0.2 g, 1 mmol), and poly(ethylene glycol) palladium (Pd/PEG) (0.8 g, 0.15 mol%) were added, and the mixture was stirred at 0 °C for 15 min. Later, a solution of 1,2-dibromoethane (2 g, 10 mmol) dissolved in 40 mL of anhydrous DMF was added dropwise, and the resulting mixture was refluxed at room temperature for 7 h. The reaction was quenched with 1 M HCl and washed to neutrality with deionized water. The inorganic phase was extracted with dichloromethane (3 × 40 mL), while the organic phase was dried over anhydrous Na_2_SO_4_. The residue was purified and recrystallized from a mixture of dichloromethane/hexane (1:1 *v/v*) to obtain a white solid powder (Figure 3). Yield: 88%. M_p_ = 120–125 °C. ^1^H NMR (400 MHz, CDCl_3_, ppm): δ 7.12 (s, 4H, Ar-**H**, J = 7.1 Hz), 4.50 (s, 4H, -C**H_2_**-C**H_2_**-, J = 5.8 Hz), 3.81 (s, 12H, Ar-O-C**H_3_**), 3.89 (s, 6H, COOC**H_3_**). ^13^C NMR (101 MHz, CDCl_3_, ppm): δ 165.1 (ester carbonyl), 153.1, 142.7, 125.0, 105.8 (phenyl ring), 69.5 (aliphatic carbon), 56.66 (methoxy carbons attached to the phenyl ring), 51.5 (ester methyl group).

#### 3.3.2. General Synthesis of Poly(ether-ester)s

In this investigation, direct esterification/copolymerization methods were applied to synthesize two series of random bio-based materials: one ester series (HA1, HB1, HC1) and one ether series (HA2, HB2, HC2). The preparation of these poly(ether-ester)s followed a two-step melt polymerization as a facile and green semi-continuous process. The synthesis setup consisted of a 250 mL steel reactor attached to a condenser, stirrer, vacuum pump, and a gas inlet. In the reactor, DEMV was added with varying amounts of aromatic diols (H), as well as CHDM in a molar ratio of DEMV:H:CHDM of 2.5:1.8:1, with Ti(OBu)_3_ (0.5% relatively to DEMV) as catalyst. This optimal molar ratio was determined through an orthogonal test. The reaction was pre-heated at 150 °C under N_2_ flow, and then the temperature was raised to 160–180 °C over a period of 4–6 h to achieve complete esterification (1st step). This process was performed under slight overpressure to enhance the release of water and methanol droplets. The vapor droplets were distilled periodically to prevent oxygen from entering the reactor. In the polymerization process (2nd step), the temperature was elevated to 200–230 °C, and the pressure was reduced to 0.05 MPa for a period of 4–5 h. This process was monitored through the stirring torque, and all reactions were stopped when the same torque value was attained. Finally, the polymers were removed from the reactor under N_2_ pressure, allowed to cool to room temperature, dissolved in chloroform and precipitated in cold methanol to purify the polymers, and dried at 50 °C under vacuum for 12 h. The yield of purified polymers varied from 82 to 91%. After drying and molding, samples were either cut into dumbbell-shaped tensile bars for testing or ground into powder for thermal analysis.

#### 3.3.3. Synthesis of Poly(1,2-diylphenyl biphenyl-co-CHDM-3,5-(bis)dimethoxyvanillate) (HA1)

A mixture of 12.5 g (15.3 mmol) of DEMV, 8.6 g (11.2 mmol) of 1,2-diethanol phenylene, 4.7 g (6.4 mmol) of CHDM and Ti(OBu)_3_ (0.5% relatively to DEMV) as catalyst were added stepwise into a 250 mL reaction bottle. The reaction mixture was heated to 180 °C under N_2_ for 4 h (1st step). Later, the polycondensation was achieved at 220 °C under vacuum and monitored through the stirring torque. After the reaction was completed, the polymer was allowed to cool to ambient temperature, dissolved in a minimum amount of CH_3_Cl, precipitated in excess methanol, isolated by filtration, and dried under vacuum to afford 20.9 g of the pure polymer (83.4% yield). ^1^H NMR (400 MHz, CDCl_3_) d (ppm) = 7.19–7.15 (dd, 4H, Ar-**H**), 6.90 (s, 4H, Ar-**H**, diester), 4.58 (t, 4H, –OC**H_2_**–CH_2_–), 4.50 (s, 4H, Ar-C**H_2_**-C**H_2_**-Ar), 3.83 (s, 12H, –OC**H_3_**), 3.12 (dd, 4H, -C**H_2_**-CH-), 2.81 (t, 4H, -CH_2_-C**H_2_**-Ar), 2.18–1.52 (m, 10H, CHDM). ^13^C NMR (101 MHz, CDCl_3_) d (ppm) = 168.9, 165.5, 145.7, 121.4, 109.8 (Ar-C, diester), 155.1, 137.9, 129.6, (Ar-C, diol), 83.3, 68.8, 41.1 (CHDM), 60.1 (O–CH_3_), 69.5, 65.1, 32.5 (aliphatic carbons).

#### 3.3.4. Synthesis of Poly(1,2-diylphenyloxy biphenyl-co-CHDM-3,5-(bis)dimethoxyvanillate (HA2)

An 11.8 g (15.1 mmol) amount of DEMV, 8.7 g (11.4 mmol) 1,2-diethyl phenylenoxy, 4.6 g (6.6 mmol) of CHDM and Ti(OBu)_3_ (0.5% relatively to DEMV) were added into a 250 mL reactor, and the temperature was set to 160 °C to complete the 1st step. In the 2nd step, the temperature was increased to 210 °C. After the reaction was completed and allowed to cool to ambient temperature, the crude polymer was purified as above to afford 21.3 g of the product (yield of 85.1%). ^1^H NMR (400 MHz, CDCl_3_) d (ppm) = 7.12 (s, 4H, Ar-**H**, diester), 6.98–6.84 (dd, 4H, Ar-**H**), 4.62 (t, 8H, Ar–C**H_2_**–C**H_2_**–Ar), 4.49 (s, 4H, –OC**H_2_**–C**H_2_**O–), 3.83 (s, 12H, OC**H_3_**–), 3.12 (dd, 4H, -C**H_2_**-CH), 2.03–1.62 (m, 10H, CHDM). ^13^C NMR (101 MHz, CDCl_3_) d (ppm) = 168.9, 163.5, 156.1, 145.7, 129.7, 122.4, 109.5 (Ar-C, diester), 153.7, 133.9 (Ar-C, diol), 83.4, 40.1, 30.6, 29.4 (CHDM), 59.1 (O–CH_3_), 72.5, 69.8, 36.2 (aliphatic carbons).

#### 3.3.5. Synthesis of Poly(1,3-diylphenyl biphenyl-co-CHDM-3,5-(bis)dimethoxyvanillate) (HB1)

An 11.9 g (15.5 mmol) amount of DEMV, 8.8 g (10.8 mmol) 1,3-diethanol phenylene, 4.9 g (7.1 mmol) of CHDM, and Ti(OBu)_3_ (0.5% relatively to DEMV) were placed in a 250 mL reactor. The reaction temperature was set to 170 ^o^C to achieve the 1st step and further increased to 220 °C to achieve the 2nd step, affording 22.1 g of purified polymer (88.2% yield). ^1^H NMR (400 MHz, CDCl_3_) d (ppm) = 7.40 (m, 1H, Ar-**H**, diester), 7.12 (s, 4H, Ar-**H**), 6.85-6.93 (m, 3H, Ar-**H**), 4.58 (t, 4H, Ar–C**H_2_**–CH_2_–), 4.49 (s, 4H, Ar–C**H_2_**–C**H_2_**–Ar), 3.81 (s, 12H, OC**H_3_**–), 3.29 (dd, 4H, O–C**H_2_**–CH), 3.10 (t, 4H, Ar–C**H_2_**–CH_2_–), 1.97–1.35 (m, 10H, CHDM). ^13^C NMR (101 MHz, CDCl_3_) d (ppm) = 166.8, 163.1, 156.3, 145.1, 128.7, 121.4, 109.8 (Ar-C, diester), 143.7, 135.6, 131.5, 127.9 (Ar-C, diol), 83.1, 69.9, 41.1, 30.4 (CHDM), 59.3 (O–CH_3_), 72.5, 66.8, 37.2 (aliphatic carbons).

#### 3.3.6. Synthesis of Poly(1,3-diylphenyloxy biphenyl-co-CHDM-3,5-(bis)dimethoxyvanillate) (HB2)

A 12.1 g (14.8 mmol) amount of DEMV, 8.5 g (11.4 mmol) of 1,3-diethanol phenylenoxy, 4.1 g (6.7 mmol) of CHDM, and Ti(OBu)_3_ (0.5% relatively to DEMV) were used. The reaction temperature was set to 165 °C to complete the 1st step, and later raised to 215 °C to complete the 2nd step, affording 20.7 g of polymer after purification (82% yield). ^1^H NMR (400 MHz, CDCl_3_) d (ppm) = 7.39 (s, 1H, Ar-**H**), 7.12 (s, 4H, Ar-**H**, diester), 6.38–6.61 (dd, s, 3H, Ar-H), 4.62 (t, 4H, –OC**H_2_**–C**H_2_**O–), 4.49 (s, 8H, Ar–C**H_2_**–C**H_2_**–Ar), 3.83 (s, 24H, OC**H_3_**–), 3.10 (dd, 4H, O–C**H_2_**–CH–), 1.75–1.35 (m, 10H, CHDM). ^13^C NMR (101 MHz, CDCl_3_) d (ppm) = 172.3, 169.7, 163.5, 156.3, 146.6, 145.7, 130.6, 122.4, 109.2 (Ar-C, diester), 163.0, 122.9, 109.1, 103.6 (Ar-C, diol), 83.5, 69.8, 41.8, 29.6 (CHDM), 59.1 (O–CH_3_), 72.5, 39.4, 66.9 (aliphatic carbons).

#### 3.3.7. Synthesis of Poly(1,4-diylphenyl biphenyl-co-CHDM-3,5-(bis)dimethoxyvanillate) (HC1)

An 11.7 g (15.0 mmol) amount of DEMV, 8.8 g (11.6 mmol) of 1,4-diethanol phenylene, 4.7 g (6.4 mmol) of CHDM, and Ti(OBu)_3_ (0.5% relatively to DEMV) were added to a 250 mL reaction flask. The temperature was set to 180 °C in the 1st step, and raised to 230 °C in the 2nd step, affording 23.0 g of purified polymer (91% yield). ^1^H NMR (400 MHz, CDCl_3_) d (ppm) = 7.12 (s, 4H, Ar-**H**, diester), 6.81 (s, 4H, Ar-**H**), 4.63 (t, 4H, Ar–C**H_2_**–CH_2_–), 4.48 (s, 8H, Ar–C**H_2_**–C**H_2_**–Ar), 3.83 (s, 12H, OC**H_3_**–), 3.12 (dd, 4H, O–C**H_2_**–CH), 2.03–1.38 (m, 10H, CHDM). ^13^C NMR (101 MHz, CDCl_3_) d (ppm) = 170.9, 163.5, 156.1, 145.7, 127.7, 121.4, 108.8 (Ar-C, diester), 138.4, 130.6 (Ar-C, diol), 83.4, 69.7, 40.2, 30.6 (CHDM), 59.3 (O–CH_3_), 72.5, 57.8, 37.5 (aliphatic carbons).

#### 3.3.8. Synthesis of Poly(1,4-diylphenyloxy biphenyl-co-CHDM-3,5-(bis)dimethoxyvanillate) (HC2)

An 11.9 g (15.6 mmol) amount of DEMV, 8.4 g (11.2 mmol) of 1,4-diethanol phenylenoxy, 4.6 g (6.5 mmol) of CHDM, and Ti(OBu)_3_ (0.125% relatively to DEMV) were loaded in a 250 mL reaction flask. The reaction temperature was set to 175 °C to complete the 1st step, and later raised to 225 °C in the 2nd step, affording 21.9 g of purified polymer (87.6% yield). ^1^H NMR (400 MHz, CDCl_3_) d (ppm) = 7.12 (s, 4H, Ar-**H**, diester), 6.98 (s, 4**H**), 4.68 (dd, 8H, Ar–C**H_2_**–CH_2_–Ar), 4.50 (s, 4H, O–C**H_2_**–C**H_2_**–O), 3.86 (s, 12H, OC**H_3_**–), 3.12 (t, 4H, O–C**H_2_**–CH), 2.12–1.38 (m, 10H, CHDM). ^13^C NMR (101 MHz, CDCl_3_) d (ppm) = 169.9, 163.5, 156.1, 145.8, 128.7, 121.4, 109.8 (Ar-C, diester), 154.1, 118.2 (Ar-C, diol), 83.4, 41.2, 30.6 (CHDM), 56.3 (O–CH_3_), 72.5, 69.4, 66.9 (aliphatic carbons).

## 4. Conclusions

In the this study, the effects of ester-ether groups and cyclic acyl chain units on the physical, thermal and mechanical properties of poly(ether-ester)s were investigated. In terms of reactivity, the ether series were more reactive than the ester series due to the inductive effect from the β-oxygen atom in aromatic ether diols. Herein, the copolymerization strategy comprised a two-step process that afforded high molecular weight materials for all series. The good thermal stability displayed by the novel symmetric monomer DEMV, derived from methyl 4-hydroxy-3,5-dimethoxybenzoate, afforded satisfactory copolymerization yields at temperatures under 230 °C. The results obtained clearly demonstrate the feasibility of these novel vanillin-based biopolymers from renewable sources (not only using DEMV, but also diols, which can be obtained via green renewable biological processes). The final morphology of the materials, free volume, crystallinity, and surface functionality could be tuned by simply varying the aromatic-aliphatic moieties used, as well as the spatial arrangement of the phenyl rings along the chains. In all cases, the weight-average molecular weight was over 50,000 g/mol, and the polydispersity indices varied from 1.54–2.88. It was shown that the poly(ether-ester)s exhibited high glass transition temperatures from 94–120 °C, depending on the side-chain length and steric hindrance on the phenyl rings (shorter side-chains led to higher T_g_ values). Additionally, their melting points varied from 205–240 °C, with heat of fusion values above 51 J/g. Mechanical testing showed that the average maximum tensile strength, elongation at break and storage modulus of the samples varied from 37–75 MPa, 750–1050% and 34.5–37 MPa, respectively. It was determined that the ether series displayed better barrier properties than the ester series, presumably due to the ability of ethers to generate hydrogen bonding. However, HC1 provided a remarkably low permeability due to its high molecular weight and symmetric structure.

The degradation of polymeric materials under different conditions has become a subject of great interest to the scientific community. Herein, the integrity of the copolymers was examined at 37 °C, including an enzymatic degradation pathway meant to simulate in vivo degradation. The effects of PP-L activity were visible on the entire polymer surface, as demonstrated by SEM analysis, and attributed to the cleavage of labile bonds. These poly(ether-ester)s were highly susceptible to enzymatic degradation (>35% weight loss) at pH 7.4 in the presence of lipase, as compared to other biobased polyesters with similar chemical structures. This property is vital for applications where the self-degradation of materials is required at the end of their life cycle. Despite all the positive scientific advances achieved so far, there is still much to do to attain a sustainable bioeconomy, including the development of renewable, time-efficient, and cost-effective methods to support, improve and replace conventional monomers. To achieve this ambitious goal, advanced investigations on the culture of new microorganisms, either from land or marine ecosystems, adapted to cost-effective plastics biodegradation, is essential to mitigate the foreseen nuisance of microplastics worldwide.

## Figures and Tables

**Table 1:** Summary of synthetic conditions and molecular weight results for poly(ether-ester)s.

Samples	HA_1_	HA_2_	HB_1_	HB_2_	HC_2_	HC_1_	DEMV-H
Diester: H: CHDM	3: 2.5: 1	3: 2.5: 1	3: 2.5: 1	3: 2.5: 1	3: 2.5: 1	3: 2.5: 1	2: 1.5
Diol	meta	meta	ortho	ortho	para	para	/
2nd step (°C) ^r^	220	210	220	215	220	230	220
Reaction time (h)	8	7.5	7	9.5	8	8	7
a ^x^	2.03	2.05	2.01	2.03	2.05	2.03	/
b ^x^	1.51	1.48	1.44	1.48	1.48	1.50	/
[η] (dL/g) ^y^	0.68	0.64	0.75	0.88	1.13	1.08	1.01
$\delta_{1,2}$ J^1/2^ cm^−3/2^	21.5	23.9	22.7	24.4	28.8	28.5	22.0
M_n_ (g/mol) ^z^	30,800	31,800	35,500	35,200	35,400	36,000	18,700
*M*_w_ (g/mol) ^z^	55,100	52,700	58,000	54,300	77,100	78,800	43,800
Ð^z^	1.78	1.65	1.63	1.54	2.17	2.88	2.31
Yield (%)	83.40	85.15	88.22	82.00	91.10	87.64	86.70

^r^ Polymerization temperature for poly(ether-ester)s. (a,b) ^x^ Mole composition of DEMV and aromatic diols relative to CHDM determined by ^1^H NMR spectroscopy. ^y^ Intrinsic viscosity; $\delta_{1,2}$ calculated solubility parameters for rigid against flexible segment; ^z^ GPC analysis in CHCl_3_ vs. polystyrene standards.

**Table 2:** Thermal properties of poly(ether-ester)s measured by TGA and DSC.

Samples	T_d,5%_ (°C)	T_d,50%_ (°C)	T_d,max_ (°C)	T_g_ (°C)	T_m_ (°C)	T_c_ (°C)	ΔH_m_ (J/g)	ΔH_c_ (J/g)	R_700_ (wt%)
HA1	410	425	465	111	215	132	77.5	−52.5	8.3
HA2	315	319/412	435	94	205	128	73.8	−51.2	8.6
HB1	420	435	470	116	230	144	85.5	−75.5	9.3
HB2	322	330/409	440	105	220	140	79.3	−71.7	8.4
HC1	430	445	485	120	240	156	105.6	−87.9	8.8
HC2	340	341/425	455	109	228	152	95.2	−86.2	8.1
PE-ms [59]	341	387	416	66					16.07
PEV [55]				83	251	132	103.6	>245	
PET [44,68]	268.9	342.8	361.7	85.4	254.9	-	31.4	4.2	

**Table 3:** Tensile properties of poly(ether-ester)s and compared with other polymers.

Polymer	Tensile Modulus (MPa)	Tensile Strength (MPa)	Elongation at Break (%)	Storage Modulus (E’, GPa)	Tan δ
PET [61,68]	1137	84.8	82.7		
PBFGA [73]	1030 ± 40	22.5 ± 0.4	285 ± 7		
PE-ms [59]	210 ± 10	5.6 ± 0.8	310 ± 30	15.7	
HC1	1580 ± 10	75 ± 0.5	1050 ± 10	38.0	68
HC2	1440 ± 10	70 ± 0.5	980 ± 10	36.5	60
HB1	1330 ± 10	65 ± 0.5	990 ± 10	36.2	63
HB2	1050 ± 10	56 ± 0.5	880 ± 10	35.1	56
HA1	980 ± 10	52 ± 0.5	950 ± 10	34.8	62
HA2	960 ± 10	44 ± 0.5	750 ± 10	34.5	60

**Table 4:** O_2_/CO_2_ Barrier properties of poly(ether-ester)s vs. PET.

Polymer ^a^	O_2_ (barrer) ^b^	BIF_PO2_^c^	CO_2_ (barrer) ^d^	BIF_PCO2_^c^
HC1	0.018 ± 0.02	0.14	0.021 ± 0.01	0.35
HC2	0.053 ± 0.02	0.41	0.048 ± 0.01	0.80
HB2	0.089 ± 0.02	0.68	0.065 ± 0.01	1.10
HB1	0.120 ± 0.02	0.92	0.067 ± 0.01	1.10
HA2	0.141 ± 0.02	1.10	0.070 ± 0.01	1.12
HA1	0.144 ± 0.02	1.11	0.075 ± 0.01	1.25
^e^ PET [55,73]	0.130 ± 0.02	1	0.060 ± 0.01	12.6
^f^ PEF [85]	0.012	73.7	0.08	63.3
DEMV-CHDM	1.01	25.4	2.77	75.3

^a^ Tests performed at low pressure (0.1001 MPa). ^b^ O_2_ permeability coefficient at 23 °C. 1 barrer = 10^−10^ cm^3^·cm/cm^2^·s·cmHg. **^c^** Barrier improvement factor (BIFp), BIF = PO_2_(PET)/PO_2_. ^d^ CO_2_ permeability coefficient. ^e^ Gas barrier properties of PET. ^f^ Gas barrier properties of PEF.

## Data Availability

The authors declare that the data supporting the findings of this study are provided in the main article and can be accessed upon request via email to the corresponding authors.
